# Iron-induced Hypophosphatemic Osteomalacia—An Atypical Case of Bilateral Femoral Stress Fractures

**DOI:** 10.5435/JAAOSGlobal-D-22-00155

**Published:** 2023-05-03

**Authors:** Kiran Kancherla, Harry Constantin, Andrew Kanawati, Edward Graham

**Affiliations:** From the Orthopaedic Department, Westmead Hospital, Sydney, Australia.

## Abstract

We present a case of a 61-year-old healthy man who had bilateral femoral neck insufficiency fractures attributed to repeated iron transfusions, causing iron-induced hypophosphatemic rickets, requiring surgical intervention. Atraumatic insufficiency fractures present a diagnostic dilemma in orthopaedics. Chronic fractures with no acute precipitating trigger can often go unrecognized until complete fracturing or displacement occurs. Early identification of the risk factors in conjunction with a comprehensive history, clinical examination, and imaging can potentially avoid these serious complications. Atraumatic femoral neck insufficiency fractures have been sporadically reported in the literature, often unilateral and attributed to the use of long-term bisphosphonates. Through this case, we elaborate on the relatively unknown link between iron transfusions and insufficiency fractures. This case highlights the importance of early detection and imaging of such fractures from an orthopaedic perspective.

Femoral neck insufficiency fractures are an uncommon injury, with bilateral fractures an even rarer occurrence (Figures 1 and 2). Femoral neck stress fractures make up 5% of all stress fractures and are often seen in athletes, elderly or those with metabolic bone disease.^1^

**Figure 1:**
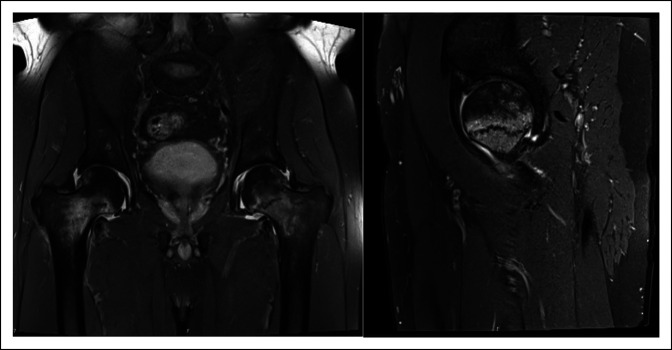
**A,** Coronal T2-weighted sequence of the pelvis and proximal femora illustrating hypointense linear signal alteration consistent with bilateral nondisplaced transverse femoral neck insufficiency fractures with associated bilateral hip effusions and (**B**) sagittal cross-section of left hip illustrating incomplete fracture of the femoral neck.

**Figure 2:**
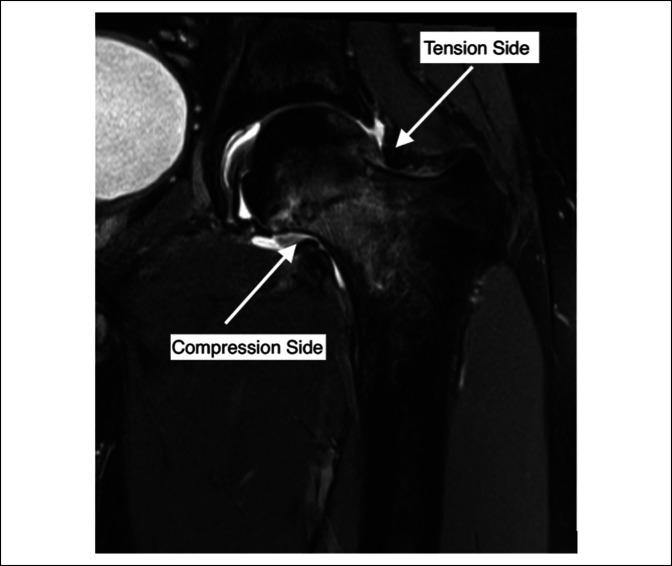
Patient's left femoral neck MRI illustrating compression side insufficiency fracture with an associated hip effusion. Given age and bilateral nature of patient's insufficiency fracture, decision was made to proceed with surgical intervention.

Normal bone formation relies on the delicate equilibrium that exists between bone synthesis and resorption. The accumulation of repetitive eccentric load through the bone with abnormal bone homeostasis leads to nonrepairable microfracturing.

The increasing availability and widespread usage of intravenous iron (ferric carboxymaltose) in the management of anemia and anemia causing conditions such as Crohn's, menorrhagia, and bowel angiodysplasia have led to the increasing occurrence of a rare but important reaction known as iron-induced hypophosphatemic osteomalacia.

We present a case of a 61-year-old man with bilateral femoral insufficiency fractures due to repeated iron transfusions, causing iron-induced hypophosphatemic rickets. The mechanism and management of these injuries will be discussed.

## Case Report

A 61-year-old man presented to hospital with a 3-month history of bilateral hip pain worse with mobilization. Initial radiographs of the hip and pelvis were normal. Subsequently performed magnetic resonance imaging (MRI) findings were consistent with bilateral nondisplaced femoral neck insufficiency fractures with associated bone marrow edema and bilateral hip joint effusions (Figure 1).

The patient had a medical history of bowel angiodysplasia and a vascular malformation in the bowel, resulting in chronic anemia. This anemia required regular iron transfusions over the preceding 8 years. The patient reported an increased rate of transfusions over the past 2 years requiring second monthly Ferinject (Ferric carboxymaltose) infusions.

Blood tests revealed hypophosphatemia, hypocalcemia, and a vitamin D deficiency. Based on the above findings, a provisional diagnosis of hypophosphatemic osteomalacia leading to insufficiency fractures was made (Table 1).

**Table 1:** Postoperative Blood Test Results Which Reveal Hypophosphatemia, Low Normal Vitamin D Levels, Normal Fibroblast Growth Factor 23 Levels, and Increased Urinary Phosphate

Test	Value	Normal Reference Range
Phosphate	0.55 mmol/L	0.75-1.50 mmol/L
24 hour urinary phosphate	49.0 mmol/24 hr	13-42 mmol/24 hr
Calcium	2.06 mmol/L	2.15-2.55 mmol/L
Fibroblast growth factor 23	53 ng/L	23.2-95.4 ng/L
25-OH vitamin D	54 nmol/L	>50 nmol/L

To mitigate any risk of these fractures completing, the patient underwent bilateral single-stage surgical fixation using a sliding hip screw construct. The postoperative course was uncomplicated.

### Discussion and Review of Literature

Atraumatic insufficiency fractures in young individuals are often an underrecognized but potentially preventable surgical condition. By the time a patient presents for an orthopaedic review with insufficiency fractures, preventive measures are often insufficient. We report a patient who presented with insufficiency fractures requiring surgical intervention secondary to hypophosphatemic osteomalacia after repeated iron transfusions.

### Diagnosis and Treatment of Hypophosphatemic Osteomalacia

The diagnosis was confirmed with blood tests that demonstrated a reduced phosphate level with increased phosphate wasting, a reduced 25-OH vitamin D level, and a normalized fibroblast growth factor 23.

Iron transfusion–induced insufficiency fractures have been noted sporadically in the literature, but there is a general paucity of research surrounding this topic. Incidence of hypophosphatemia has been noted in up to 86% of patients after iron transfusions but are noted to mostly be transient and clinically insignificant in the patient group.^2^

It is thought that ferric carboxymaltose infusion therapy causes an increase in serum levels of fibroblast growth factor 23 by inhibiting FGF 23 degradation.^3^ FGF 23 is a glycoprotein produced by osteocytes and osteoblasts in response to high serum phosphate levels, leading to the down regulation of NaPi2a and NaPi2c expression in the brush border of the proximal tubule,^4^ resulting in increased phosphate wasting. FGF 23 further suppresses the activity of 1-α hydroxylase in kidneys to decrease the formation of 1,25-dihydroxyvitamin D, a precursor of 25-hydroxyvitamin D.^5^ Bone mineralization is ultimately suppressed by hypophosphatemia and low serum 25-hydroxyvitamin D, leading to the formation of insufficiency fractures as seen in this case.^6^

In this case, an FGF 23 level of 53 was taken to be abnormal in the context of chronic hypophosphatemia because one would expect low levels of FGF 23 through negative feedback. Above normal levels of urinary phosphate and low normal 25-hydroxyvitamin D further support the diagnosis of iron transfusion–induced osteomalacia. Postoperatively, the patient was treated with phosphate, vitamin D, and calcium replacement.

### Mechanism of Femoral Neck Insufficiency Fractures

The femoral neck can be stressed because of the anatomical loading forces through the bone on movement.^7^ On axial loading, bending force is used repeatedly, and thus, the femoral neck is more easily broken in minor trauma in the patient with osteomalacia.^8^ Owing to the lack of cortical breach (a complete fracture), insufficiency fractures are often too subtle to be detected on radiographs. A high index of suspicion and early use of MRI imaging may improve the success rates of detection of this process.

Insufficiency fractures of the femoral neck were first broadly differentiated in 1964—separated radiologically into transverse (tension) and compressive type fractures.^9^ Transverse fractures are typically found in the elderly population involving the superior-lateral aspect of the neck and are most at risk of complete fracture if left unmanaged. Compression-type fractures, as seen in our patient, often involve the inferior-medial femoral neck in a younger population group and less often proceed to complete displacement. Fullerton et al^10^ further expanded this classification system in 1988 to include displaced fractures as the third part of the classification system. With the advent and widespread uptake of MRI and bone scan imaging, earlier recognition of insufficiency fractures was made appreciable. In 1997, Arendt et al^11^ further differentiated insufficiency fractures from grade 1 to 4, taking into account positive early findings on MRI (Table 2).

**Table 2:** Grading of Insufficiency Fractures Taking Into Account MRI and Radiograph Findings as per the Arendt Stress Fracture Severity Scale

Grade	STIR* Signal Change	T2 Signal Change	T1 Signal Change	Plain Radiograph
1	Positive	Negative	Negative	Negative
2	Positive	Positive	Negative	Negative
3	Positive	Positive	Positive	Periosteal reaction
4	Positive	Fracture line noted	Fracture line noted	Fracture line noted

* Short T1 inversion recovery imaging.

### Surgical Intervention

**Figure 3:**
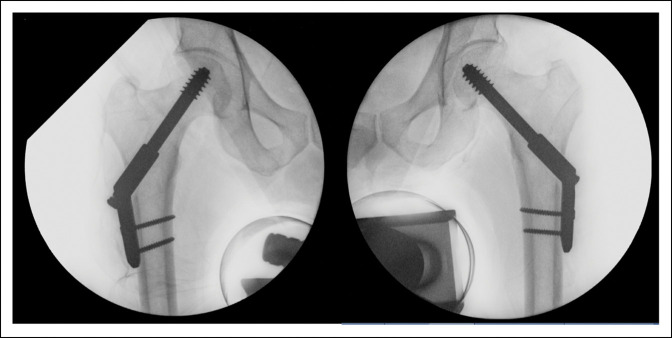
Patient's intraoperative images illustrating bilateral two-hole dynamic hip screw fixation.

Once diagnosed, the circumstances and point under which a patient should have an operation present an additional dilemma. Fullerton described compression side insufficiency fractures with a fatigue line <50% of femoral neck width as nonsurgical injuries—managed with non–weight bearing, crutches, and activity restriction. Tension side fractures and with a fatigue line >50% were further described as requiring surgical fixation.^10^ Building on this, a 2018 retrospective study found that those patients with a fracture line on initial MRI with an associated hip effusion had eight times the risk of fracture progression as compared with those without an effusion and as such should be considered for early surgical intervention.^12^ In our case, although the patient had bilateral compression type fractures, the patient's MRI reported bilateral hip effusions, and given that maintaining bilateral non–weight status in an otherwise healthy patient would be undesirable, the decision was made to proceed to surgical fixation. Figure 2 shows preoperative MRI findings. Figure 3 shows postoperative imaging.

## Conclusion

We report a 61-year-old man who presented to our hospital with bilateral femoral insufficiency fractures on the background of repeated iron transfusions for bowel angiodysplasia. We aim to emphasize that a high index of suspicion, low threshold to proceed to MRI, surgical intervention, and endocrinology referral are warranted for patients with a similar presentation.
